# Extended Lichtenstein Repair for an Additional Femoral Canal Hernia

**DOI:** 10.3390/jcm13185386

**Published:** 2024-09-11

**Authors:** Johan De Gols, Evelien Berkmans, Mieke Timmers, Cedric Vanluyten, Laurens J. Ceulemans, Nathalie P. Deferm

**Affiliations:** 1 Department of Abdominal Surgery, Sint-Franciscus Hospital, 3550 Heusden-Zolder, Belgium; johan.degols@sfz.be (J.D.G.); evelien.berkmans@sfz.be (E.B.); mieke.timmers@sfz.be (M.T.); nathalie.deferm@sfz.be (N.P.D.); 2 Department of Thoracic Surgery, University Hospitals Leuven, 3000 Leuven, Belgium; cedric.vanluyten@uzleuven.be; 3 Laboratory of Respiratory Diseases and Thoracic Surgery (BREATHE), Department of Chronic Diseases and Metabolism (CHROMETA), KU Leuven, 3000 Leuven, Belgium

**Keywords:** femoral hernia, hernia repair, herniorrhaphy, inguinal hernia, Lichtenstein repair

## Abstract

The Lichtenstein procedure is one of the most performed surgeries worldwide. However, proper examination to exclude a femoral hernia is often not performed, resulting in a high number of missed hernias. For patients in whom a femoral hernia is suspected pre- or intraoperatively, we describe a novel surgical technique of a femoral extension to the classic Lichtenstein repair. We aim to investigate its safety and clinical outcome. **Methods:** The femoral–extended Lichtenstein is applied when a femoral hernia is suspected. The fascia transversalis is opened, the lacunar ligament incised, and the hernia reduced. A self-gripping mesh covers the femoral orifice equally on all sides. In a prospective single-center study, we compared 50 consecutive femoral–extended to 50 classic Lichtenstein repairs, evaluating operative time, patient-reported pain (intensity, duration), and recurrence. **Results:** The technique seems feasible and safe. Apart from 3 min additional surgical time, no difference in pain scoring or hernia recurrence was observed between both groups. **Conclusions:** We successfully introduced a femoral-extended Lichtenstein repair for patients with suspected femoral herniation.

## 1. Introduction

The lifetime risk for developing groin herniation has been estimated at 27% for men and 3% for women; thus, groin hernias remain one of the most common surgical pathologies worldwide [1]. Generally, groin hernias are categorized into inguinal or femoral, depending on the protrusion arising either above or below the inguinal ligament. A femoral canal hernia occurs through a space bounded superiorly by the iliopubic tract (Poupart’s ligament), inferiorly by the pectineal ligament (Cooper’s ligament), laterally by the femoral vein, and medially by the insertion of the iliopubic tract into Cooper’s ligament. While femoral hernias account for less than 10% of all groin hernias, they are relatively more common in women over the age of 70 [2,3]. Due to the high risk of complications, elective repair is recommended. Nevertheless, 40% of femoral hernias present as an emergency with incarceration and/or strangulation requiring bowel resection in up to 23% of the cases, significantly higher compared to 0.6% after elective femoral hernia repair [2,4].

Surgical hernia repair is performed as either an open or laparoscopic procedure. Open procedures are generally divided into two types: a tension-free repair using a mesh (e.g., Lichtenstein) or a sutured repair (e.g., McVay). A study by Waltz et al. suggested that in 13% of all groin hernia repairs, a femoral hernia was detected in addition to or instead of the inguinal hernia [5]. Furthermore, 61% of patients with a femoral hernia underwent previous open inguinal hernia repair. This can indicate either the development of a femoral hernia over time or failure to recognize a concomitant femoral hernia during initial open repair.

In the classic Lichtenstein technique, a polypropylene mesh is placed between the inguinal floor and the external oblique muscle’s aponeurosis. This mesh eliminates the need for tension sutures and avoids using weakened tissues for inguinal hernia repair. When intra-abdominal pressure rises during physical exertion, the contraction of the external oblique muscle creates counterpressure on the mesh, allowing the increased pressure to assist in the repair process [6]. Although the classic Lichtenstein repair is not applicable to femoral hernias, since the femoral ring is not covered, the more elaborate McVay repair closes the femoral canal and is therefore suitable for when an additional femoral hernia is encountered. However, the latter is characterized by a high risk of recurrence due to tension at the suture line.

To properly treat a femoral hernia, we report a novel surgical technique of a femoral extension to the classic Lichtenstein repair. This technique could decrease the recurrence rate after groin hernia repair.

## 2. Methods

In this single-center prospective study, the surgeon investigated each elective groin hernia repair for whether a femoral hernia was present. If it was diagnosed prior to or exposed during hernia repair, patients underwent the femoral–extended Lichtenstein technique and were allocated to the ‘femoral group’. From December 2017 until January 2021, 50 consecutive patients were accordingly treated and included. 

As a control group, 50 consecutive patients with a non-femoral inguinal hernia were included from August 2021 to February 2022 and treated by standard Lichtenstein repair. Patients with concomitant epigastric, umbilical, or incisional hernias were excluded, as were patients with cryptorchidism. Patients lost to follow-up and/or who did not fill in the questionnaires were also excluded. The final date of follow-up was 31 December 2022. This study was approved by the local ethical committee of the Sint-Franciscus Hospital, Heusden-Zolder. Informed consent was obtained from each patient prior to surgery.

Data were collected prospectively. Demographics included age, sex, weight, height, body mass index (BMI), presence of diabetes mellitus, smoking status, and hernia laterality and type. Outcomes were defined as duration of surgery, hospital stay, patient-reported outcome, and recurrence. Surgical time was measured as the interval between the incision and skin closure. Outcomes on pain intensity and duration were evaluated using patient-filled questionnaires at three weeks postoperatively. Pain intensity was graded according to a visual analogue scale (VAS) on a 0 to 10 numeric rating, where 0 indicates ‘no pain’ and 10 ‘pain as bad as it could possibly be’. Duration of pain was measured as the number of days after surgery during which the patient experienced pain. Hernia recurrence was defined by a positive radiological evaluation using ultrasonography when clinical examination was suspicious.

Statistical analysis was performed using GraphPad Prism 9. Continuous variables are reported as means (± standard deviation (SD)); nominal variables are presented as absolute numbers or frequencies (%). Continuous and nominal variables were compared using Student’s *t*- and Fisher’s exact tests. *p*-values < 0.05 were considered statistically significant.

### 2.1. Surgical Procedure

**Figure 1:**
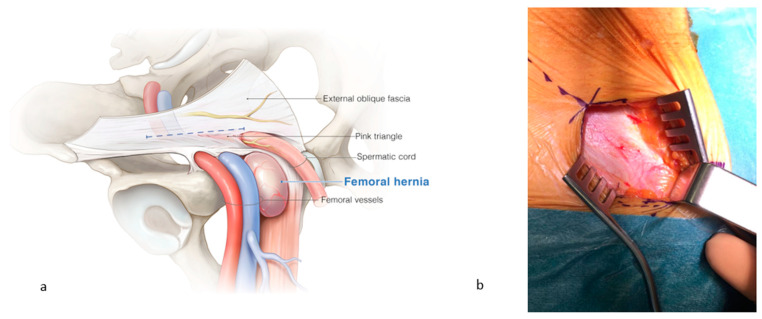
(**a**) A femoral hernia is localized at the medial side of the femoral vessels, caudal to the inguinal canal. (**b**) Incision of the external oblique fascia, just above the ‘pink triangle’, formed by the diversion of the vessels of this fascia towards the external inguinal orifice. ©spMedical-illustration—produced for JDG.

All procedures were performed under general anesthesia by a single surgeon (J. De Gols). All patients received standard prophylactic antibiotics (one dose of cefazolin) 30 min prior to the incision. After opening of the skin and subcutis, the external oblique fascia was incised just above the ‘pink triangle’, formed by the diversion of the vessels of this fascia towards the external inguinal orifice (Figure 1). This is important to keep enough fascia caudally for the fixation of the two self-gripping meshes. The femoral region was explored by digital palpation. The pubic bone is located medially, the femoral vein and pulsating artery laterally, the inguinal ligament of Poupart ventrally, and the pectineal ligament of Cooper is on the dorsal side. Digital palpation with the fingertip may reveal bulging but not herniation. When it is difficult to localize the surrounding structures correctly, one must be aware of the presence of a femoral hernia, since the herniating tissue will cover the surrounding structures.

**Figure 2:**
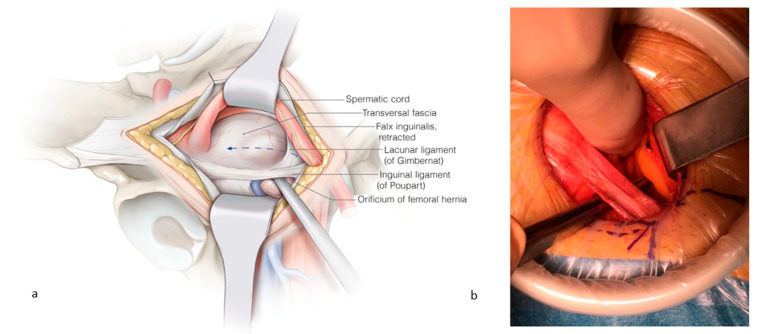
(**a**) The spermatic cord is retracted cranially, and the fascia transversalis is opened from medial to lateral to avoid bleeding from the femoral vessels. The instrument is placed through the femoral hernia orifice. (**b**) The lacunar ligament is incised in order to create space for the placement of the femoral mesh. ©spMedical-illustration—produced for JDG.

When a femoral hernia is palpated, the fascia transversalis is opened from medial to lateral in order to avoid bleeding from the femoral vessels. Cooper’s ligament is freed. The lacunar ligament is incised to create enough space for the mesh (Figure 2). Sometimes, small veins require clipping at the dorsal border of Cooper’s ligament. The femoral hernia is reduced, and the femoral vein becomes visible at the lateral side.

### 2.2. Mesh Preparation

All femoral hernias were repaired using the Parietal ProGrip self-gripping meshes (Medtronic). This is a self-gripping prosthesis made from synthetic material. The prosthesis is cut to size: 7 cm from ventral to dorsal and 4.5 cm from medial to lateral. In one case, a bigger mesh, namely 7 to 5.5 cm, was used to cover a major femoral hernia.

### 2.3. Mesh Placement

**Figure 3:**
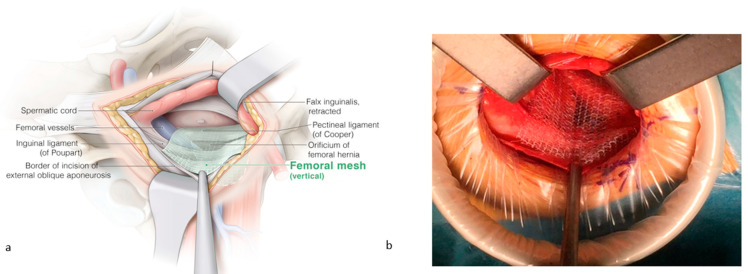
(**a**) The femoral mesh is placed over Cooper’s ligament and folded over it mainly dorsally and a bit caudally. The remaining mesh covers the femoral orifice with an equal overlay medially over the insertion of the pectineal ligament and laterally over the femoral vessels. (**b**) Ventrally, the mesh is approaching the incisional border of the external oblique fascia. ©spMedical-illustration—produced for JDG.

The falx inguinalis is retracted upward to free Cooper’s ligament. The mesh is placed over Cooper’s ligament and folded over it mainly dorsally and a bit caudally, with the self-gripping side towards the ligament. The remaining mesh covers the femoral orifice with an equal overlay medially over the insertion of the pectineal ligament and laterally over the femoral vessels. Ventrally, the mesh is approaching the incisional border of the external oblique fascia (Figure 3). The femoral orifice is now covered. Suturing is not required.

**Figure 4:**
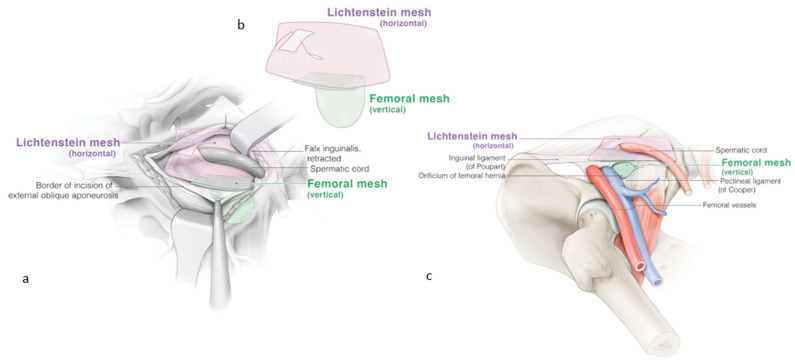
(**a**) Reinforcement of the inguinal region with a classic Lichtenstein repair, the pink mesh. The femoral extension of the Lichtenstein technique with the secondary green mesh for the femoral hernia. (**b**) The self-gripping prosthesis for the femoral mesh is cut to size: 7 cm from ventral to dorsal and 4.5 cm from medial to lateral. (**c**) The final result with both meshes, preferably with an overlap of approximately one centimeter. ©spMedical-illustration—produced for JDG.

The inguinal region is reinforced with the same self-gripping mesh according to the Lichtenstein technique. The caudal border of the Lichtenstein mesh ends at the same position as the ventral border of the femoral mesh, preferably with an overlap of approximately one centimeter (Figure 4). The external oblique fascia is closed without tension to avoid additional postoperative pain. Scarpa’s and Camper’s fascia are closed. The skin is sutured. No drainage is required.

## 3. Results

In each group, 45 patients with a unilateral and 5 patients with a bilateral hernia were included. The mean age in the femoral hernia group was 59 years (± 15), comparable to the control (i.e., non-femoral inguinal hernia) group (61 ± 15; *p* = 0.436). In the femoral group, 30% of the patients were female, which was significantly higher compared to 2% in the non-femoral group (*p* < 0.001). There were no differences in weight, height, or BMI, nor in the presence of diabetes mellitus or active smoking status. All demographics are summarized in Table 1.

Surgical time was longer (29 vs. 26 min; *p* = 0.044) in patients with a unilateral femoral hernia compared to a non-femoral hernia (Table 2). For both groups, the mean hospital stay was one day and most of the patients were discharged on the day of the surgery. Pain intensity did not differ between both groups and patients were pain-free after an average period of 5 days. Two patients in the femoral group reported signs of nerve entrapment (superficialis cutaneous nerve) which resolved after single infiltration with xylocaine 2%. There were no wound-related complications and in both cohorts, no recurrence was observed. The mean follow-up of the femoral group was 32 months (±12) and the mean follow-up of the non-femoral group was 12 months (±3).

## 4. Discussion

In this report, we introduced and visualized an innovative surgical technique that extends the classic Lichtenstein repair to simultaneously address femoral canal hernias. Our findings suggest that this technique is both feasible and safe, as evidenced by our comparison of 50 consecutive extended procedures for femoral herniations with 50 consecutive classic Lichtenstein repairs for non-femoral herniations. The extended procedure required only an additional 3 min of surgical time, with no significant differences in short- or long-term clinical outcomes observed between the two groups. This technique can be used for all femoral hernias. 

The literature indicates that the incidence of femoral hernias is four times higher in women [2,3]. In our study, 30% of patients in the femoral–extended cohort were female, a significantly higher proportion compared to the non-femoral group. While ultrasound is an excellent tool for diagnosing inguinal hernias, it often fails to differentiate between various hernia locations. Our findings suggest that intraoperative digital palpation is a reliable method for identifying femoral hernias, as no femoral hernias were diagnosed during the follow-up of the control group. Therefore, we propose reserving the extended technique for cases where a femoral hernia is diagnosed pre- or intraoperatively.

The development of a groin hernia carries both short-term and long-term risks. While watchful waiting may be appropriate for men with minimal symptoms, untreated inguinal hernias can lead to severe complications, such as bowel incarceration or strangulation. Emergency repair of inguinal hernias significantly increases the risk of mortality, which is twice as high compared to elective procedures [7]. Since femoral hernias are more likely to incarcerate and strangulate, timely hernia repair in women with groin herniation is recommended [8]. 

Regarding long-term risks, a recent review of 25 studies involving 6293 participants reported that 2–4% of patients experience hernia recurrence after repair, depending on whether mesh was used, and 5–10% suffer from chronic postoperative pain [9]. In our cohort, the use of a self-gripping mesh was a key element, as it eliminated the need for suturing in the lateral region where the femoral vessels are located. This suture-avoiding approach likely reduces the risk of postoperative seroma, hematoma, and pain [10]. Furthermore, with the use of a self-gripping mesh, operative time was significantly reduced compared to the conventional suture fixation approach [11]. Patient-reported outcomes on pain intensity and duration were similar between both groups. Except for two patients in the femoral group who experienced nerve entrapment of a superficialis cutaneous nerve and required a single infiltration with xylocaine 2%, neither group reported any chronic lasting postoperative pain.

Although the laparoscopic approach is often considered superior for inguinal hernia repair due to its ability to easily identify and treat femoral hernias, especially in women, open inguinal hernia repair remains one of the most frequently performed surgical procedures worldwide. This is particularly true in regions where laparoscopy is not available. Our study found no recurrence of hernias, suggesting that thorough digital exploration of the groin can effectively exclude the presence of femoral hernias. 

Our study is limited by its single-center and non-randomized design. However, all procedures were performed by an experienced abdominal wall surgeon. To further validate the reproducibility and effectiveness of this technique in preventing recurrence after inguinal hernia repair, larger multi-center studies involving different surgeons are needed.

## 5. Conclusions

We successfully introduced a new technique to treat a femoral hernia during an open groin hernia procedure. In comparison to a classic Lichtenstein repair for non-femoral hernia, there was no difference in postoperative pain intensity or duration. No recurrence was observed.

## Figures and Tables

**Table 1:** Demographics and risk factors.

		Femoral Hernia (N = 50)	Non-Femoral Inguinal Hernia (N = 50)	*p*-Value
**Age** (years)	Mean (± SD)	59.06 (± 15.31)	61.46 (± 15.40)	0.436
**Sex**				
Female	n/N (%)	15/50 (30.0%)	1/50 (2.0%)	**< 0.001**
Male	n/N (%)	35/50 (70.0%)	49/50 (98.0%)	
**BMI** (kg/m^2^)	Mean (± SD)	25.40 (± 3.18)	26.45 (± 4.27)	0.168
**Weight** (kg)	Mean (± SD)	76.42 (± 11.66)	82.20 (± 18.07)	0.060
**Height** (cm)	Mean (± SD)	173.32 (± 8.52)	175.66 (± 7.58)	0.150
**Diabetes mellitus**				
No	n/N (%)	49/50 (98.0%)	45/50 (90.0%)	0.092
Yes	n/N (%)	1/50 (2.0%)	5/50 (10.0%)	
**Active smoker**				
No	n/N (%)	40/50 (80.0%)	33/50 (66.0%)	0.115
Yes	n/N (%)	10/50 (20.0%)	17/50 (34.0%)	
**Side**				
Unilateral	n/N (%)	45/50 (90.0%)	45/50 (90.0%)	1.00
Bilateral	n/N (%)	5/50 (10.0%)	5/50 (10.0%)	

Legend: SD, standard deviation; BMI, body mass index; kg, kilogram; cm, centimeter.

**Table 2:** Perioperative outcome, pain assessment, and recurrence.

	Statistic	Femoral Hernia (N = 50)	Non-Femoral Inguinal Hernia (N = 50)	*p*-Value
**Duration (minutes)**				
Unilateral (*n* = 45)	Mean (± SD)	28.71 (± 5.78)	26.33 (± 5.25)	**0.044**
Bilateral (*n* = 5)	Mean (± SD)	60.80 (± 5.72)	56.00 (± 14.37)	0.507
**Hospitalization** (days)	Mean (± SD)	1.06 (± 0.24)	1.16 (± 0.51)	0.212
**Pain** (VAS)	Mean (± SD)	5.10 (± 2.48)	4.66 (± 2.53)	0.382
**Pain** (days)	Mean (± SD)	5.40 (± 4.08)	5.34 (± 4.13)	0.942
**Recurrence**				
No	n/N (%)	50/50 (100.0%)	50/50 (100.0%)	1.00
Yes	n/N (%)	0/50 (0.0%)	0/50 (0.0%)	

Legend: SD, standard deviation; VAS, visual analog scale.

## Data Availability

The data presented in this study are available on request from the corresponding author, ‘the data are not publicly available due to privacy or ethical restrictions.
